# Healthcare provision for displaced people in transit: Analyses of routinely collected data from INTERSOS clinics at the Ukrainian border with Moldova and Poland

**DOI:** 10.1016/j.jmh.2024.100287

**Published:** 2024-12-22

**Authors:** Saleh Aljadeeah, Seyed-Moeen Hosseinalipour, Nataliia Khanyk, Eszter Szocs, Aliki Traianou, Ana Tomas, Chrysanthi Tatsi, Elżbieta Czapka, Alessandro Verona, Tessa van Boekholt, Ion Chesov, Apostolos Veizis

**Affiliations:** a Department of Public Health, Institute of Tropical Medicine, Antwerp, Belgium; b Geneva Centre of Humanitarian Studies, Faculty of Medicine, University of Geneva, Switzerland; c Pharmacoepidemiology & Social Pharmacy, Department of Pharmacy, Uppsala University, Sweden; d Department of Pharmacy, Danylo Halytsky Lviv National Medical University, Ukraine; e Mental Health Program, International Academy Arts-Culture & Formation, Italy; f School of Medicine, University of Glasgow, UK; g Faculty of Medicine, University of Novi Sad, Serbia; h Ärzte der Welt e.V (Doctors of the World), Dnipro, Ukraine; i Sociology Department, University of Gdańsk, Poland; j INTERSOS, Italian Society of Migration Medicine, Italy; k INTERSOS Moldova, Chisinau, Moldova; l Nicolae Testemitanu State University of Medicine, Chsinau, Moldova; m INTERSOS HELLAS, Thessaloniki, Greece

**Keywords:** Refugees, Conflict, Ukraine, Transit, Border, Access to healthcare

## Abstract

**Background:**

The Russian military invasion of Ukraine has sparked Europe's largest forced displacement since World War II, bringing about significant health vulnerabilities for migrants and refugees. European health information systems lack comprehensive data coverage, especially in underrepresented migration stages like transit. This study aims to address this gap by analyzing data from INTERSOS clinics at the Moldovan and Polish borders with Ukraine to identify the common health conditions prompting people to seek healthcare services during transit.

**Methods:**

From 6th March to 31st May 2022, this cross-sectional study observed migrants and refugees receiving INTERSOS services at two mobile clinics in Moldova, and a facility in Poland. We analyzed data by age, sex, nationality, and reported disease frequencies and care provided.

**Results:**

This study used routinely collected data from 1756 patients. The majority of the adult population seeking healthcare were females (76.5 %), 26.1 % were children and 18.7 % were older individuals. Noncommunicable diseases (NCDs) were the primary reason for seeking healthcare at these clinics, with 23.3 % of the study population being diagnosed with at least one chronic disease, and 3.4 % of people having multimorbidity. Mental and behavioural disorders were diagnosed in 12.6 % of the population, and somatoform disorders and related stress (F40-F48) in 10.8 %.

**Discussion:**

Our study indicates the diversity of forcibly displaced populations and the corresponding diversity of their healthcare needs. In the shadow of a forced displacement crises triggered by the conflict in Ukraine, there is an urgent need to give more attention to subgroups of the population that are often neglected in humanitarian crises. These include older adults, females, and ethnic minorities. Additional attention should also be given to NCDs and sexual and reproductive health (SRH) care needs, especially given the high numbers of older adults and females in this population. Early medical support, psychological first aid and interventions in transit centers that promote survivor resilience and recovery are required.

**Conclusion:**

Our findings shed light on the healthcare needs of forcibly displaced populations during transit, a stage often overlooked in migration health research. They underscore the diverse healthcare needs of forcibly displaced populations, emphasizing the necessity for humanitarian aid programs to acknowledge and address this diversity. Accessing data on displaced populations' healthcare needs in transit can help the preparing and planning of healthcare services for these populations in host or destination countries. Ensuring objective anonymization and preventing patient re-identification are essential, particularly in safeguarding refugee privacy and data protection to avoid the misuse of their data.

## Background

The Russian military invasion of Ukraine has triggered Europe's fastest and largest forced displacement since World War II. As of February 2024, 3.7 million people were internally displaced people in the Ukraine, and 6.5 million refugees from Ukraine have been recorded across Europe, according to the United Nations High Commissioner for Refugees (UNHR) (UNHCR 2024; European Union 2022). Forced displacement creates new health vulnerabilities and negatively affects the outcomes of pre-existing health conditions (Aljadeeah et al., 2022, Continuum of care for noncommunicable disease management during the migration cycle 2022). Migrants' and refugees' access to healthcare in reception countries is limited due to several factors, including legal restrictions, knowledge of entitlement, language barriers, and health-seeking behaviours (S Aljadeeah et al., 2021).

Different countries have varying policies and approaches when providing health and social protection services depending on the country's legal framework, the number of migrants and refugees within its borders, its economic capacity, and its political climate. In 2022 in Moldova, refugees have free access to emergency and primary care but this does not include access to medicines and secondary care (UNHCR 2023). In Moldova compulsory health insurance has been jointly funded by the state and individuals since 2004 (UNHCR 2023). This foundational policy was put to the test during the forced displacement emergency in February 2022, when Moldova committed to covering emergency healthcare costs for all refugees. By June 2022, the Moldovan government extended its healthcare services to cover all refugee children. These services include prophylactic exams, child development monitoring, vaccinations, primary care, and specialized hospital services. (UNHCR 2023).

In Poland, accessing health care services requires the PESEL (Polish acronym for Universal Electronic Population Registration System) number (European Union Agency for Asylum (euaa) 2022). The PESEL number is assigned to children born in Poland when they are issued their birth certification by a Polish civil registry office. It includes children of Polish citizens, citizens of the European Union (EU) and members of their families who live in Poland, and who have obtained the right of permanent residence. It also includes those with refugee status, subsidiary protection, those with asylum claims, permits for tolerated stay temporary protection, and permission to stay for humanitarian reasons. Medical examination is free for Ukrainian refugees in Poland but the cost of medicines is not covered (European Union Agency for Asylum (euaa) 2022). From February 24, 2022, refugees from the Ukraine who had not obtained asylum status or temporary protection could apply for a PESEL number giving them the same access to services as Polish citizens, including vaccinations as well as social and health assistance[7].

Language and cultural barriers are often obstacles for migrants and refugees seeking access to health and social protection services in host countries, as they hinder effective communication and understanding between service providers and those in need (S Aljadeeah et al., 2021). The Russian language is widely spoken in Moldova and the Ukraine, and they have strong cultural ties which results in less need for cultural-linguistic mediation. In Poland, cultural- linguistic- mediation was underestimated and deemed unnecessary, given the similarities between the Polish and Ukrainian languages.

In response to the forced displacement crisis in Ukraine, International Non-Governmental Organizations (INGOs) are playing an essential role in providing humanitarian assistance, including healthcare services, to those people feeling into neighbouring countries (UNHCR 2023). Routinely collected data from INGOs can serve as valuable evidence to answer questions related to access and use of healthcare service and contribute to improved decision-making in refugee crisis management. INTERSOS, an international humanitarian organization established in Italy, provides essential assistance, protection, psychological first aid, and medical care to populations affected by conflict, disaster, and extreme poverty (INTERSOS 2024). In the Ukraine-Russia conflict, INTERSOS has supported Ukranian migrants and refugees offering healthcare and protection services at the border with Poland since February 26, 2022, and in Moldova from March 2, 2022. The data provided by this organization will be utilized in this research.

European health information systems lack comprehensive and high-quality data coverage regarding migrants and refugees (Bozorgmehr et al., 2016, S Aljadeeah et al., 2021). There is a need to improve the existing data on health status, needs, and access to healthcare, to tailor care to the affected population's needs (Lebano et al., 2020). The lack of availability of routine data such as patient health data, vaccination records, and information on access to health care, is a barrier to designing and implementing effective services and equitable access to health and social care (World Health Organization 2023). There is an increasing need for representative health data (Aljadeeah, 2022).

Migration involves various stages, including departure, transit, arrival, settlement, and return or deportation (European Union 2022). No single data source can fully track the health of this it is crucial to consider the diverse migration stages and trajectories (Bozorgmehr et al., 2023, S World Health Organization 2020), especially in underrepresented migration stages, such as transit (European Union 2022). During the transit stage, migrants can be exposed to a variety of challenges, including poor living conditions and exposure to violence and exploitation, which will impact on their health across all the stages of migration (European Union 2022, Lebano et al., 2020). There is a significant gap in the literature about access to healthcare in the transit stage of the migration cycle (European Union 2022, World Health Organization 2022). This study aims to answer the following research questions: What are the most common reasons for seeking healthcare services among the displaced population in the transit stage, and what are their unmet healthcare needs in this stage? To answer these questions, we used routinely collected data from INTERSOS clinics at the borders with Moldova and Poland.

## Materials and method

### Setting and data sources

This study employed a cross-sectional design to report data on migrants and refugees who received INTERSOS services at two mobile clinics in Moldova, and at a medical facility in Poland from March 6 to May 31, 2022. INTERSOS, is a non-governmental organization (NGO) that provides healthcare services in humanitarian settings, and collects and manages data on these services(INTERSOS 2024). The study provides a snapshot of the healthcare needs of migrants and refugees at a specific period of time. This design is particularly suitable for identifying the percentage of various health conditions and the demographic characteristics of the affected population (Setia, 2016). Other study designs, such as longitudinal studies, were deemed less feasible due to the high mobility and temporary status of migrant populations in transit.

The INTERSOS mobile medical clinic at the Palanca Bus Transit Centre in Moldova provided a package of services including healthcare and protection assistance (legal, child protection, gender-based violence protection, psychosocial support, and information dissemination). People that crossed the southern border of Moldova by foot were brought here before boarding buses to their next destination, either Romania or Chisinau. The medical services mainly focused on first aid, the stablilization of acute cases and primary health care. For emergency presentations an ambulance was called using the local system which could take up to 40 min to arrive from the nearest hospital. INTERSOS also offered primary healthcare services at one of the refugee accommodation centres in the southern part of Moldova. In Poland, the INTERSOS medical point had six small private rooms for the examination and the management of cases and one emergency room with a defibrillator, an electrocardiogram and oxygen.

During healthcare visits at INTERSOS clinics, a range of variables were captured for each migrant and refugee. These variables included: i) sociodemographic features: (age, sex, and nationality) ii) the frequencies and percentage of diseases that led to seeking healthcare services: (infectious diseases, and non-communicable diseases (NCDs), iii) vaccination status, iv) vulnerabilities, and v) healthcare interventions. For this study, we utilized a selection of these variables to address our research questions. Missing data was managed by analyzing the available data for each variable. For each analysis, only cases with complete data for the variables of interest were included. The extent of missing data for each variable was documented, and these missing values were excluded from the specific analyses.

The data was collected through the KoBo Toolbox platform; the diagnoses were recorded following the International Classification of Diseases (ICD-10). For data protection, the UNHCR monitoring tool was used, as UNCHR is an INTERSOS’ partner in Poland and Moldova. Vulnerabilities were identified through the UNHCR protection-monitoring tool, and disabilities identified through the Washington questionnaire. Coordination activities with the National Healthcare Systems in Poland and Moldova were developed bilaterally at the district level through the national coordination group facilitated by the Ministries of Health and WHO, as well as in the operational coordination of emergency medical teams led by the WHO.

### Data analyses

Our study focused on the following outcomes: i) sociodemographic features: (age, sex, and nationality) ii) the frequencies and percentage of diseases that led to seeking healthcare services: (infectious diseases, and non-communicable diseases (NCDs), iii) vaccination status and iv) vulnerability. The data was described or analyzed by variables of age, sex, and nationality. We reported the frequencies and percentages of diseases that led to seeking healthcare services for the total study population, and by age and sex. We also presented the interventions provided to the study population.The diagnoses reported in the data were coded according to the ICD system. Data was analysed using Mirosoft Excel (2021).

### Ethical consideration

The study used routinely collected data. The data was anonymised to ensure there were no details that could lead to the identification of individuals within our study population. The data for our analysis was provided by INTERSOS. The required Institutional Review Board approvals were obtained from the Nicolae Testemițanu State University of Medicine and Pharmacy, Moldova (18.05.2023/Nr 5–30), and the University of Gdańsk, Poland (18.04.2023), ensuring ethical compliance throughout our research process.

## Results

The study used routinely collected data from 1756 patients from INTERSOS clinics at the Ukrainian borders with Moldova and Poland. Of the total population, 70.7 % (1242) were females, 26.1 % (458) were <18 years old, and 18.7 % (328) were older than 65 years. (Table 1).

**Table 1:** Characteristics of the study sample by age, sex and location.

Category	n	%
**Adults**		
**Sex**		
Female	993	56.6
Male	303	17.3
Missing	2	0.1
**Age**		
18–64	970	55.2
65+	328	18.7
Missing	0	0
**Country of medical intervention**		
Moldova	610	34.7
Poland	666	37.9
Missing	22	1.2
**Children**		
**Sex**		
Female	249	14.2
Male	203	11.6
Missing	2	0.1
**Age**		
0–5	201	11.4
6–17	254	14.5
Missing	0	0
**Country of medical intervention**		
Moldova	158	9
Poland	282	16.1
Missing	15	0.9
**Total**	**1.756**	**100**

The majority of the of the study population were Ukrainian nationals (87.5 %), 3.3 % were Moldavian nationals, 2.6 % were Uzbek nationals and 6.6 % were other nationalities. A total of 747 patients (42.54 %) declared their final destination country. The most popular countries were Moldova (38.82 %), Germany (14.59 %), Poland (11.11 %), Romania (7.49 %), and Italy (3.74 %) (Supplementary Table 1).

Fifty-four cases of injuries connected with the conflict were identified. All of these were caused by damage from shrapnel or glass from explosions and were mainly superficial wounds. The most seriously injured were evacuated directly to Rzeszow airport and then relocated to neighbouring or other European countries. In Poland there were about 8000 daily arrivals during the first few weeks of the war and generally they had minor medical needs. From April 2022, the number of arrivals reduced significantly, but the number of medically and socially critical and complex cases increased.

The most common diagnoses were conditions affecting the cardiovascular system (17.3 %), digestive system (15.8 %) and mental and behavioural disorders (12.6 %) (Table 2). Partial data was available on mental health conditions of the study population which were diagnosed in 12.6 % of the population (ICD-10 class V), and somatoform disorders and related stress (F40-F48) in 10.8 %. Of the total population 70.9 % are female, and 28.8 % are male. The most represented age group is 18–49 years (34.77 %), followed by the 50–64 age group (20.42 %), and 18.7 % are over 65. Patients under 18 years old were 25.9 % of the total population with 14.5 % aged 6–17 years, and 11.4 % aged 0–5 years.

**Table 2:** Macrodiagnoses.

Macrodiagnosis	Female		Male		Total	
	n	%	n	%	n	%
IX Diseases of the circulatory (cardiovascular) system	250	14.2	54	3.1	304	17.3
XI Diseases of the digestive system	174	9,9	103	5.9	277	15.8
V Mental and behavioural disorders	195	11.1	26	1.5	221	12.6
X Diseases of the respiratory system	109	6.2	58	3.3	167	9.5
N/A	109	6.2	44	2.5	153	8.7
XIII Diseases of the musculoskeletal system and connective tissue	80	4.6	38	2.1	118	6.7
I Certain infectious and parasitic diseases	56	3.2	29	1.6	85	4.8
XII Diseases of the skin and subcutaneous tissue	50	2.8	32	1.8	82	4.7
VI Diseases of the nervous system	42	2.4	21	1.2	63	3.6
IV Endocrine, nutritional and metabolic diseases	38	2.1	17	1.0	55	3.1
XIX Injury, poisoning and certain other consequences of external causes	28	1.6	26	1.5	54	3.1
XVIII Symptoms, signs and abnormal clinical and laboratory findings, not elsewhere classified	31	1.8	20	1.1	51	2.9
vii diseases of the eye and adnexa	18	1.0	17	1.0	35	2
XIV Diseases of the genitourinary system	23	1.3	9	0.5	32	1.8
VIII Diseases of the ear and mastoid process	13	0.7	7	0.4	20	1.1
II Neoplasms	9	0.5	3	0.2	12	0.7
XV Pregnancy. childbirth and the puerperium	8	0.5	0	0	8	0.5
XX External causes of morbidity and mortality	4	0.2	2	0.1	6	0.3
XXI Factors influencing health status and contact with health services	3	0.2	1	0	4	0.2
XVII Congenital malformations. deformations and chromosomal abnormalities	1	0.1	3	0.2	4	0.2
III Diseases of the blood and blood-forming organs and certain disorders involving the immune mechanism	2	0.1	1	0.1	3	0.2
Missing	2	0.1	0	0	2	0.1
**Total**	1245	70.9	551	29.1	1756	100

**Fig. 1:**
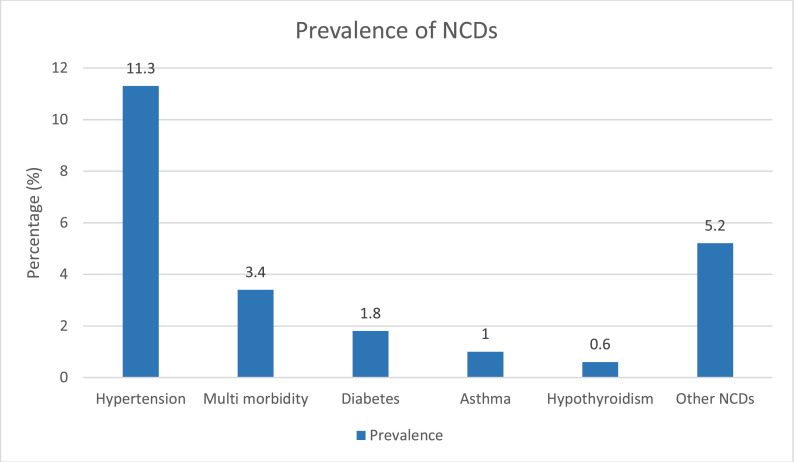
Proportion (%) of NCDs.

A total of 409 (23.3 %) people were diagnosed with at least one chronic disease, and 60 (3.4 %) people were identified with multimorbidity. Hypertension and diabetes were the most commonly diagnosed NCDs among the study population (Fig. 1).

About 10 % of people (181) were diagnosed with at least one infectious disease, and half of them had acute upper respiratory infections (Table 3). Thirteen people (0.7 %) tested positive for COVID -19, while 356 (20.3 %) tested negative. The remaining 1387 people (79.0 %) were not tested for COVID -19.

**Table 3:** Reported infectious diseases in the study population.

Infectious diseases	n	%
Acute upper respiratory infections (J00-J06)	93	5.3
Other infectious diseases (B99-B99)	43	2.5
Intestinal infectious diseases (A00-A09)	21	1.2
Other viral diseases (B25-B34)	9	0.5
Mycoses (B35-B49)	6	0.3
Viral infections characterized by skin and mucous membrane lesions (B00-B09)	3	0.2
Other bacterial diseases (A30-A49)	2	0.1
Lung diseases due to external agents (J60-J70)	2	0.1
Influenza and pneumonia (J09-J18)	2	0.1
**Total**	181	10.3

**Fig. 2:**
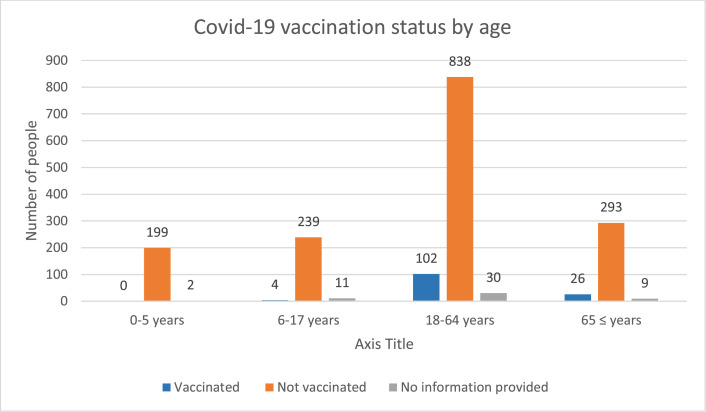
COVID-19 vaccination status by age.

A total of 1230 people (70.1 %) received at least one medication. The data did not include further information about these medications. 187 people (10.6 %) stated that they had not received any vaccines previously. One hundred and thirty-two patients (7.5 %) were vaccinated against COVID -19. One hundred and two patients (5.8 %) aged 18–64 years old were vaccinated against COVID-19, which is the largest age group of vaccinated people. 1569 (89.35 %) patients were not vaccinated against COVID-19. The COVID-19 vaccination status of 52 people (2.96 %) was not recorded (Fig. 2).

Amongst vulnerable populations eighty-one people (4.6 %) included in the study were elderly, 23 people (1.3 %) had a physical disability, and 76 people (4.3 %) had other vulnerabilities (Table 4).

**Table 4:** Reported vulnerabilities in the study population.

Vulnerability	n	%
Elderly in need of care	81	4.6
Other	76	4.3
Woman alone	60	3.4
Physical disability	23	1.3
Mental disability	15	0.9
PTSD	9	0.5
Unaccompanied Asylum Seeking Children	2	0.1
Gender-Based Violence survivor Woman alone	1	0.1
**Total**	267	15.2

## Discussion

The quantitative data analyses in this study describes the healthcare needs of a population fleeing the Ukrainian conflict at the transit stage, a stage of the migration cycle that is often underrepresented in migrants’ health research (European Union 2022). To our knowledge, this is the first study to report the common presentations that led people fleeing war in Ukraine to seek healthcare services at entry point clinics of an international NGO, at the borders with Moldova and Poland (Kumar et al., 2022). The majority of the adult population (76.5 %) were female. Of the total population who sought healthcare at these clinics, 26.1 % were children, and 18.7 % were older people. NCDs were the most common reason for accessing services. The majority of the study population (78.1 %) did not provide any information regarding their vaccination status, while only 32 out of 455 children were fully vaccinated according to national children's vaccination plan.

Our study used routinely collected data at the transit stage of the migration cycle. Notably, data on the healthcare needs of refugees at the transit stage is underrepresented (European Union 2022). Health information systems in Europe exhibit incomplete coverage of migrant and refugee populations (Bozorgmehr et al., 2023). This gap does not stem from a lack of knowledge or technology but is influenced by various political and ethical concerns hindering its resolution (Bozorgmehr et al., 2023). It is argued that the disaggregation of health data by migratory status could lead to discrimination against migrants and refugees, especially in times of increased anti-migration.

Within the European context, it is argued that current data protection regulations may restrict the disaggregation or analysis of data focusing on migrant populations due to the sensitivity of such data under the General Data Protection Regulation (GDPR) (Bozorgmehr et al., 2023, EUR-Lex 2016). These regulations, however, allow for the collection and analysis of such data under specific circumstances, provided there are valid justifications and adequate safeguards in place. Therefore, current data protection laws do not prohibit the collection and analysis of data regarding migrant and refugee health (Bozorgmehr et al., 2023, S World Health Organization 2020). There is a compelling rationale for disaggregating and analysing data focusing on migrant populations to better understand their specific health needs and challenges. Conversely, restricting the use of this data risks overlooking the unique needs of migrant populations, potentially leading to inadequate responses that do not take their needs into consideration and that further exacerbate inequalities (Bozorgmehr et al., 2023, Rechel et al., 2012, Migration in West and North Africa and across the Mediterranean 2020, Chiesa et al., 2019, Juárez et al., 2019). Ethical considerations regarding the collection, processing, storage, analysis, and dissemination of data focusing on migrant populations should be carefully addressed. Objective anonymisation and prevention of patient re-identification are crucial, with safeguarding refugee privacy and data protection being of utmost importance to prevent potential misuse of this data for purposes other than health, such as immigration enforcement (Bozorgmehr et al., 2023, S World Health Organization 2020, Migration in West and North Africa and across the Mediterranean 2020).

The majority of the study population are female. This reflects and aligns with the gender distribution in the population that has fled the war in Ukraine, where the majority of this displaced population were females, making this forced migration emergency different from other forced migration flows in the past, which involved a majority of male refugees (OECD 2023). Between 2015–2017, Eurostat data indicates that approximately 30 % of all asylum applications were submitted by females, and 35 % of all positive first-instance decisions in the EU states being granted to them (OECD 2023)**.** This could partly be explained by the compulsory military service obliging adult males to stay in Ukraine (European Union 2022).

Female refugees are more vulnerable to sexual and gender-based violence during different stages of the migration cycle (European Union 2022). Previous research has indicated that refugee women may face what has been described as a "triple disadvantage," where the intersection of gender, migration status, and forced migration exacerbates challenges, amplifying their impact through mutual reinforcement (OECD Social 2018)**.** In addition to this "triple disadvantage", it's noteworthy that in humanitarian settings, there has been scant attention given by policy, practice, and research to sexual and reproductive health (SRH) (Chynoweth et al., 2018). Neglecting SRH needs within humanitarian settings has significant repercussions. These include increased rates of preventable maternal and newborn morbidity and mortality, the avoidable aftermath of sexual violence including unintended pregnancies, and the consequences of these such as unsafe abortions, increased incidence of sexually transmitted infections (STIs), increased HIV transmission, and mental health issues, including depression and trauma (The Inter-Agency Working Group on Reproductive Health in Crises (IAWG) 2018). People affected by conflict or disaster are entitled to access to SRH care. Providing comprehensive, high-quality SRH services in humanitarian settings requires a multi-sectoral, integrated approach including protection, health, nutrition, education, water, sanitation and hygiene (WASH) and community service personnel. To ensure that SRH interventions and services meet the needs and address the cultural norms of displaced populations, meaningful community involvement should be considered in every research and intervention development phase (Aljadeeah, 2022, The Inter-Agency Working Group on Reproductive Health in Crises (IAWG) 2018).

NCDs were the most common reason for this population to seek healthcare services at the border clinics. 409 (23.3 %) people were diagnosed with at least one NCD. In Ukraine, the burden of NCDs is the highest compared to other health conditions (Murphy et al., 2022). About 33 % have hypertension, and about 7 % have diabetes (Murphy et al., 2022, World Bank 2018, S Aljadeeah et al., 2021). The management of NCDs usually requires lifestyle changes and, very often, pharmacological therapies (S Aljadeeah et al., 2021, Wirtz et al., 2016). These therapies, particularly for diabetes, can be costly for populations affected by humanitarian crises (S Aljadeeah et al., 2021). Healthcare during humanitarian crises has usually given lower priority to NCDs (S Aljadeeah et al., 2021, Khan et al., 2019). Among forcibly displaced populations, treatment of existing NCDs may be disrupted during their forced displacement and by restrictions and barriers that limit their access to healthcare services and medications in transit and on arrival in the destination countries (European Union 2022).

About 19 % (328) of the patients included in this study were over 65 years old. Older people typically experience a higher burden of disease compared with younger people (Piotrowicz et al., 2022), and they are at higher risk of frailty. The experience of forced displacement may exacerbate their vulnerability (14). Elderly refugees often face many barriers to accessing healthcare and are often given low priority in humanitarian settings. A survey among elderly Ukrainian refugees in Moldova found that 28 % urgently need different medicines, including NCD medicines and pain relief. Over a third of them cannot afford to buy these medicines (20).

While it's acknowledged that older individuals face heightened vulnerability during humanitarian crises, they have not been prioritised for humanitarian aid. Nevertheless, the practical implementation of these recommendations remains constrained in real-world scenarios. Considering the relatively high number of older people who fled the war in Ukraine, urgent attention should be given to the particular healthcare needs of this population; this includes ensuring access to housing with elderly-friendly infrastructure and maintaining continuity of care for pre-existing diseases. According to the UN Decade of Healthy Ageing 2020–2030 action plan (Armocida et al., 2022), health systems must: (1) prioritize age-inclusive emergency preparedness, response, and recovery; (2) offer integrated care for older individuals, including community support; (3) provide targeted training for healthcare personnel; and (4) ensure access to long-term care. Aligning with the humanitarian principles of humanity and impartiality, and promoting the idea of leaving no one behind and achieving the Sustainable Development Goal of healthy lives for all ages, it's crucial to recognize and leverage the resilience, strengths, and potential of older people in humanitarian settings (Armocida et al., 2022, S World Health Organization 2020).

Mental and behavioural disorders were diagnosed in 12.6 % of the population, and these disorders were more prevalent in females. Mental health problems are one profound consequence of conflict. Before the current conflict, about 30 % of the population suffered mental diseases disorders (Seleznova et al., 2023). Pre-existing mental health conditions and previous traumas may be exacerbated by grief, chronic stress, and high levels of anxiety experienced during the recent, devastating invasion. Women and children account for >90 % of the displaced, are at risk of sexual violence, rape, and trafficking during displacement. Early medical support and psychological first aid, and interventions in transit centers that promote survivor resilience and recovery are required (Stark et al., 2022). A trauma-informed assessment of the possible presence or emergence of significant mental illness is a key component and must be integrated into an overall care plan (Kaufman et al., 2022). Some diagnoses may also have revealed underlying mental health conditions (e.g., tension headache, acute hypertension). Given the dynamic nature of clinical environments characterised by urgency and instability, we anticipate that the prevalence of mental health disorders could be even greater.

The conflict in Ukraine has brought the attention to various manifestations of racism, which involved the unjust belief in the inferiority of individuals based on their skin color or ethnicity (Cénat et al., 2022). For instance, Roma refugees encounter greater obstacles in accessing assistance compared to non-Roma refugees due to discrimination and antigypsyism. This difficulty extends to accessing information, housing, employment, and healthcare services, exacerbating their sense of marginalisation and isolation. In Poland, although healthcare for refugees is theoretically free upon registration, language barriers and antigypsyist attitudes often impede access. There have been reported cases of tuberculosis and HIV patients struggling to obtain essential medicines. Adequate linguistic and cultural mediation services are not consistently provided for minority groups, including the Roma community and some members of the Asian community. Advocating for improved healthcare access, including interpreting services for conflict-affected individuals, regardless of nationality, ethnicity, age, or gender, is imperative (Mirga-Wójtowicz et al., 2022).

Tracking the national vaccination schedule and the percentage of individuals who received these vaccinations, based on their age, is vital for monitoring vaccination progress in 2021 and 2022. For instance, Ukrainian children should receive three doses of Hepatitis B vaccination before they reach 6 months of age. In 2021, 78.8 % of children who were in the 6-month age range received this vaccination. However, this percentage decreased to 37.6 % in 2022 (Holt, 2024, State institution 2024). The same pattern applies to all vaccinations. The reasons for such substantial decreases in vaccination rates can be explained by the internal and cross-border displacement, and lack of trust in governments and healthcare systems. The present study found lower rates of vaccinations in general and lower rates of “confirmed” COVID-19 vaccination in among the patients. The low rates of “confirmed” COVID-19 vaccination among patients in the over 65 age group is concerning since this group is at higher risk of Covid-19 related hospitalisation, especially for those staying in refugee reception centers.

## Strengths and limitations

To the best of our knowledge, this is the first study to report the health needs of people fleeing Ukraine in the transit stage, a stage of the migration cycle that is understudied in the literature concerning refugee healthcare needs[4]. Our analysis provides an example of how data can be collected at the transit stage of the migration cycle and used to inform the preparedness of health systems in reception countries during high influxes of forcibly displaced people. Study limitations include covering a limited period of data collection, and a possibility of inaccurate coding of conditions due to time constraints.

## Conclusions

In summary, the data reported in this study provides information about the healthcare needs of forcibly displaced populations from the Ukraine during the transit stage of migration, which is often understudied in the literature. The data indicates the diversity of the forcibly displaced populations, and the corresponding diversity in their healthcare needs which should be reflected and considered by humanitarian aid programming. In the shadow of a forced displacement crises triggered by the conflict in Ukraine, there is an urgent need to give more attention to subgroups of the population that are often neglected amid humanitarian crises. A notable finding is the high number of older adults and females within this population, underscoring the need for targeted healthcare interventions. Local response plans should address the specific healthcare needs of these groups by ensuring access to comprehensive healthcare services that cater to these needs, which should include the development of age- and gender-sensitive health programs. . Additional attention should also be given to NCDs, SRH and mental health care needs. Accessing data on displaced populations healthcare needs in the transit stage can aid efforts in healthcare delivery planning in the countries of destination or host countries. Ensuring objective anonymisation and preventing patient re-identification are essential, particularly in safeguarding refugee privacy and data protection to avoid the misuse of this data.

## Consent for publication

Not applicable

## Funding

This research did not receive any specific grant from funding agencies in the public, commercial, or not-for-profit sectors.

## Declaration of competing interest

The authors declare that they have no known competing financial interests or personal relationships that could have appeared to influence the work reported in this paper.

## Data Availability

Data is available on reasonable request.

## Appendix Supplementary materials

Image, application 1
