# Supplementary material for: Healthcare provision for displaced people in transit: Analyses of routinely collected data from INTERSOS clinics at the Ukrainian border with Moldova and Poland
Source: J Migr Health. 2024 Dec 22;11:100287. doi: 10.1016/j.jmh.2024.100287 (PMC11762559; doi:10.1016/j.jmh.2024.100287)
Supplement: Supplementary file 1 [file mmc1.docx]

**Supplementary Table 1:** countries of destination

| **Country of distention** | **n** | **%** |
| --- | --- | --- |
| Moldova | 299 | 17.0 |
| Germany | 109 | 6.2 |
| Poland | 83 | 4.7 |
| Romania | 56 | 3.2 |
| Italy | 28 | 1.6 |
| Denmark | 24 | 1.4 |
| France | 18 | 1.0 |
| Czechia | 16 | 0.9 |
| Other EU Country | 13 | 0.7 |
| Spain | 13 | 0.7 |
| N/A | 9 | 0.5 |
| Ukraine | 8 | 0.5 |
| Other countries | 81 | 4.6 |
| **Total** | 757 | 43.1 |
